# Segmental testicular infarction: a case report

**DOI:** 10.1186/s13256-017-1308-1

**Published:** 2017-05-18

**Authors:** Tine Smets, Gina Reichman, Dirk P. J. Michielsen

**Affiliations:** 1 Vrije Universiteit Brussel (VUB), Campus Jette, Laarbeeklaan 103, 1090 Brussels, Belgium; 2 Department of Urology, University Hospital Brussels (UZ Brussel), Laarbeeklaan 101, 1090 Brussels, Belgium

**Keywords:** Segmental testicular infarction, Scrotal pain, Testicular torsion, Testicular tumor

## Abstract

**Background:**

Segmental testicular infarction is a very rare condition, which can mimic a testicular torsion or testicular cancer. Correct diagnosis is difficult but it is important to avoid unnecessary radical treatment.

**Case presentation:**

We report a clinical case of a 36-year-old white man who presented at our emergency department with subacute testicular pain. A urine analysis, Doppler ultrasound, surgical exploration, blood analysis, and magnetic resonance imaging were performed to diagnose his condition, to exclude a testicular torsion, and to raise confidence in its non-malignancy. He was treated conservatively. At follow-up, a few months after the incident, he no longer had complaints. Ultrasonography showed remaining hypo-echogenicity of the left upper pole, indicating a sequel of ischemia.

**Conclusions:**

Segmental testicular infarction is a rare condition which can be easily confused with a testicular torsion or a testicular tumor. This case report can be helpful in recognizing and diagnosing this condition. Making the right diagnosis is important since it can prevent an unnecessary radical treatment.

## Background

Segmental testicular infarction is a very rare condition which can be diagnostically challenging. The etiology and pathophysiology are not well understood. Patients are mostly affected between the second and fourth decade [1]. It can mimic a testicular torsion or a testicular cancer. Many authors will opt for a radical treatment because of diagnostic uncertainty. However, with reassuring test results and watchful waiting, this can often be avoided.

## Case presentation

A 36-year-old white man with no relevant medical history presented to our emergency department (ED) with severe left scrotal pain.

Left testicular discomfort had been present for a month, and the pain had worsened in the past few hours. A scrotal examination revealed a painful and lightly swollen upper pole of his left testicle. He experienced no fever or dysuria and urine analysis was normal. Blood analysis showed a slightly elevated C-reactive protein (CRP; 13.4 mg/L), creatine kinase (368 U/L), lactate dehydrogenase (LDH; 664 U/L), and white blood cell count (WBC; 12.1×10^3^/mm^3^; 83% neutrophils). Viral epididymitis was suspected and therapy with oral anti-inflammatory drugs was started. He was advised to wear tight underpants and could return home.

**Fig. 1:**
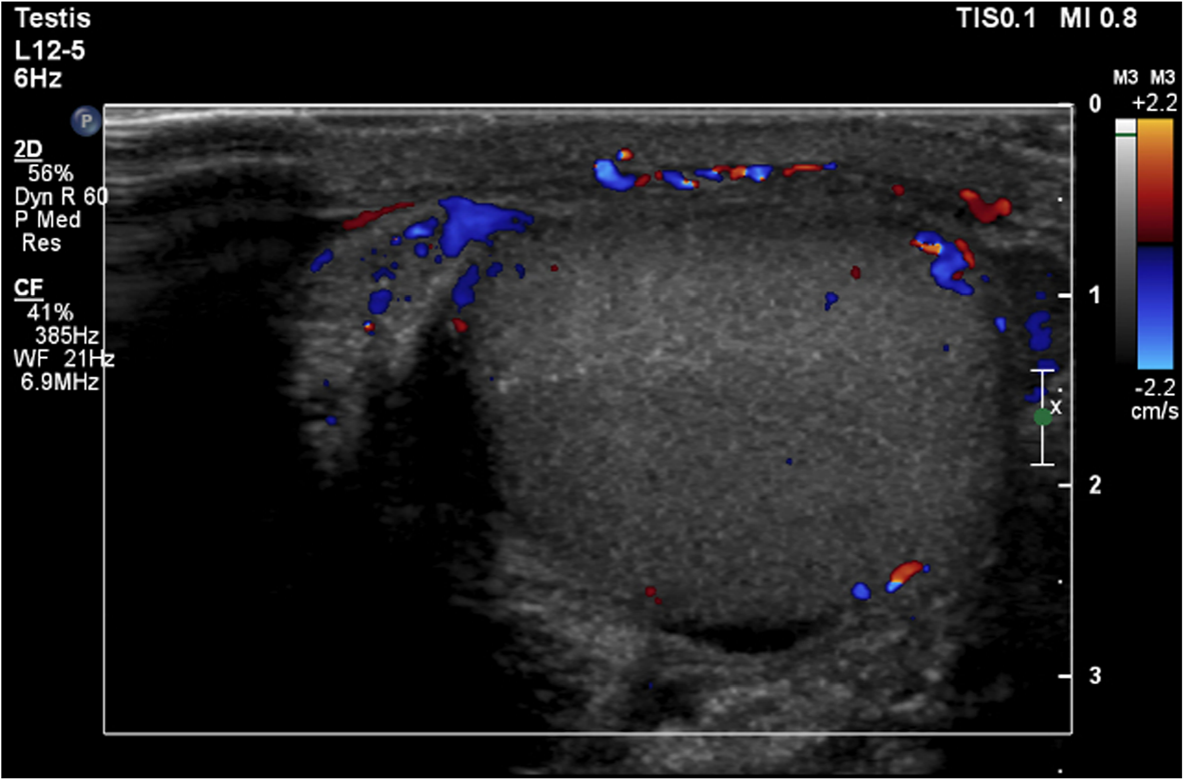
Color Doppler ultrasound of the left testicle demonstrating reduced vascularization of the left testicle

The next day, however, he returned to our ED with aggravated testicular pain and swelling despite painkillers and anti-inflammatory medication. On physical examination, no high-riding left testicle was found. Palpation of his abdomen was normal. His WBC (15.1×10^3^/mm^3^) and CRP (16.4 mg/L) had risen further. A color Doppler ultrasound evaluation revealed a reduced vascularization of his left testicle (Fig. 1); his right testicle appeared to be normal.

Surgical exploration was carried out to rule out a testicular torsion. During exploration, no torsion was found but the left testicle showed a white discoloration with a faint purple aspect at the upper pole, indicating a segmental diminished vascularization. His testicle was spared and a bilateral orchidopexy performed. Two days after surgery, his pain was under control and he was discharged from our hospital with painkillers, anti-inflammatory drugs, and anti-emetic drugs (paracetamol 1 g 4×/day, diclofenac 75 mg 2×/day, domperidone 10 mg 3×/day).

**Fig. 2:**
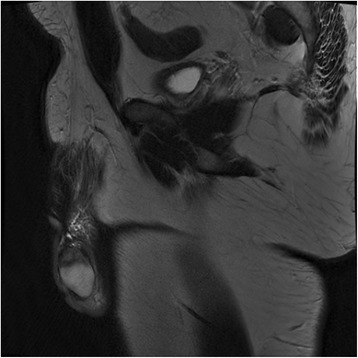
Contrast-enhanced magnetic resonance imaging, T2-weighted images showing a heterogenic hypointensity in the upper part of the testicle

A month after the operation a contrast-enhanced magnetic resonance imaging (MRI) was performed. T2-weighted images showed a heterogenic hypointensity in the upper part of his left testicle (Fig. 2), which appeared ischemic after contrast enhancement.

Tumor markers were also determined: human chorionic gonadotrophin (β-HCG), alpha fetoprotein (AFP), and LDH. Except for a slightly elevated LDH (698 U/L), the results were in the normal range. These results increased confidence in the diagnosis of segmental testicular infarction and made the hypothesis of testicular cancer less likely.

**Fig. 3:**
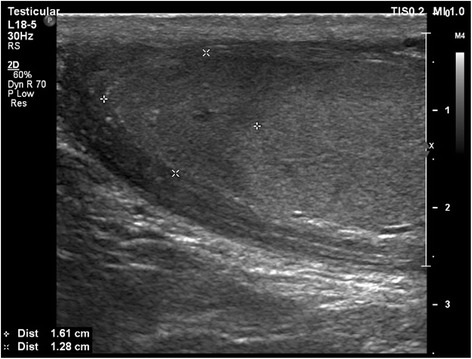
Ultrasonography showing a remaining hypofixation of the upper pole at follow-up

At follow-up a few months after the incident, our patient no longer had complaints although ultrasonography showed a remaining hypo-echogenicity of the left upper pole, indicating a sequel of the ischemia (Fig. 3).

In Table 1, all events that occurred during this case are summarized and provided in chronological order.

**Table 1:** Timeline

March 2015	Start of testicular discomfort
April 2015	Presented first time at emergency department, after blood and urine analysis he was sent home
April 2015, next day	Presented second time at emergency department: blood analysis, Doppler ultrasound, and surgical exploration; suspicion of segmental testicular infarction
May 2015	Tumor markers determined and magnetic resonance imaging performed; more certainty about diagnosis
July 2015	Follow-up ultrasonography: remaining hypofixation of left upper pole

## Discussion

In contrast to infarction of the entire testicle, segmental testicular infarction is rare and in 70% of cases no underlying cause is observed. The most common underlying cause is thought to be epididymo-orchitis [2]. This could also have been the cause in our case as our patient presented a more subacute pain which suddenly aggravated.

The pathophysiology of segmental testicular infarction is not completely understood; however, some mechanisms have been proposed. The testicle has a triple arterial blood supply: the testicular artery arising from the abdominal aorta, the cremasteric artery, and the artery of ductus deferens [3]. In some men, a segmental area of the testicle can functionally be considered an end organ. An infarction will occur in these areas if the blood flow through an end artery is interrupted and collateral blood supply is insufficient. This blood flow obstruction is mainly secondary to venous thrombosis [4].

Segmental testicular infarction is most frequently accompanied by acute scrotal pain and swelling. Our patient presented with a rather atypical subacute onset of testicular pain, but swelling was present. Gianfrilli *et al*. [5] states that this pain and swelling can be variable or even absent in some cases, leading to an incidental diagnosis. Abdominal pain can be present too. Palpation of the testicle is usually normal; however, an induration can be felt in more advanced cases [6].

An important differential diagnosis with segmental testicular infarction is a testicular tumor. Tumor markers are elevated in 60% of cases of testicular cancer. In our case we determined tumor markers β-HCG, LDH, and AFP to diminish doubts about malignancy. Only LDH was slightly elevated probably due to tissue loss caused by the segmental infarction. One must remark that a negative marker level does not prove the absence of a tumor and additional examinations are advised. Examination possibilities are: color Doppler ultrasound, contrast-enhanced ultrasonography (CEUS), and MRI [7]. Ultrasound is the examination of choice when a patient presents with acute scrotal pain.

A case study of Madaan *et al*. presenting the ultrasounds of 19 cases of segmental testicular infarction showed a hypoechoic lesion in 74%, mixed echogenicity in 21%, and a hyperechoic lesion in 5% of the cases [8]. The lesions were described as being wedge-shaped, rounded, or consisting of multiple foci with absent or low blood flow [8].

If a focal lesion is seen which is markedly less vascularized than the surrounding normal tissue, shows no mass effect, nor signs of infiltration of vascular structures or scrotal tunics [7], it is likely to be benign [2]. Further examination to exclude a hypovascular tumor is, however, necessary [7]. Horstman *et al*. described six out of seven testicular tumors smaller than 1.6 cm to be hypovascular on color Doppler ultrasonography [9].

When a discrepancy between clinical and ultrasonographical signs is present, MRI can provide extra information [10, 11]. T2-weighted images typically show a hypodense area with absence of enhancement except for the rim. On contrast-weighted T1-weighted images a segmental testicular infarction may appear isointense or show hemorrhagic foci of high signal intensity [12]. A MRI was performed in our case showing a heterogenic hypointensity (16×22×12 mm) in the upper part of our patient’s left testicle, which appeared ischemic after contrast enhancement.

Two approaches are possible in the management of segmental testicular infarction: a surgical approach or a conservative approach. Because the clinical and radiological presentation of testicular tumors and segmental testicular infarction can be similar [13], segmental testicular infarctions are often managed by performing a radical orchiectomy. Hence, a proper diagnostic investigation is indispensable to prevent such radical treatment when unnecessary.

Exclusion of testicular tumor or abscess is not always possible with MRI [10]. Therefore some authors recommend testicular exploration and biopsy when a patient presents with an indistinct lesion [14]. Ruibal *et al*. even suggest that any hypoechoic testicular lesion should be considered malignant unless proven otherwise and consider infarction to be a clear-cut indication for partial orchiectomy [4]. In contrast, Fernández-Pérez *et al*. advocate that reassuring imaging combined with negative tumor markers is sufficient for the diagnosis of segmental testicular infarction; therefore claiming that conservative follow-up, by means of watchful waiting, is reasonable [11].

In our case a surgical exploration was carried out to rule out a testicular torsion. After the exploration, a conservative approach was selected because the blood tests and imaging were reassuring.

## Conclusions

Segmental testicular infarction is a rare condition which can be easily confused with a testicular torsion or a testicular tumor. In cases similar to the one described by us, a partial or radical orchiectomy was often performed because they feared a testicular tumor. Making the right diagnosis is important in preventing this unnecessary radical treatment.

In our opinion a color Doppler sonography, followed by blood tests and MRI is sufficient for diagnosis. When followed by watchful waiting, we consider this approach justifiable and safe.

## Abbreviations

AFP: Alpha fetoprotein
β-hCG: Human chorionic gonadotrophin
CEUS: Contrast-enhanced ultrasonography
CRP: C-reactive protein
ED: Emergency department
LDH: Lactate dehydrogenase
MRI: Magnetic resonance imaging
WBC: White blood cell count
